# Mitochondria-Targeted Antioxidant MitoQ Improves In Vitro Maturation and Subsequent Embryonic Development from Culled Cows

**DOI:** 10.3390/ani14202929

**Published:** 2024-10-11

**Authors:** Zhihao Feng, Junsong Shi, Jiajie Ren, Lvhua Luo, Dewu Liu, Yongqing Guo, Baoli Sun, Guangbin Liu, Ming Deng, Yaokun Li

**Affiliations:** 1 College of Animal Science, South China Agricultural University, Guangzhou 510642, China; zhihaofeng@stu.scau.edu.cn (Z.F.); dwliu@scau.edu.cn (D.L.); yongqing@scau.edu.cn (Y.G.); baolisun@scau.edu.cn (B.S.); gbliu@scau.edu.cn (G.L.); dengming@scau.edu.cn (M.D.); 2 Yunfu Sub-Center of Guangdong Laboratory for Lingnan Modern Agriculture, Yunfu 527300, China; junsongstone@stu.scau.edu.cn (J.S.); 976991864@stu.scau.edu.cn (J.R.); luolvhua@wens.com.cn (L.L.)

**Keywords:** MitoQ, IVM, bovine oocytes, mitochondria, antioxidant, oxidative stress

## Abstract

**Simple Summary:**

There are many cull cows that have a very rich oocyte resource that can be utilized wisely to retain the high genetic value genes of the cows. However, the in vitro maturation (IVM) potential of bovine oocytes from cull cows is low. MitoQ is an antioxidant with potent effects on mitochondria. Previous studies have reported that MitoQ has a certain preventive and therapeutic effect on diseases related to the imbalance of the antioxidant system, oxidative stress, and mitochondrial dysfunction. Therefore, this study investigated the effects and mechanisms of MitoQ on the IVM of culled bovine oocytes and subsequent embryonic development. The results showed that the addition of low concentrations of MitoQ to IVM medium had a positive effect on oocyte maturation and subsequent embryonic development, maintaining normal mitochondrial function and reducing oxidative stress. In conclusion, MitoQ improved mitochondrial dysfunction, increased mitochondrial activity during IVM, and reduced oxidative stress, resulting in increased IVM rates and subsequent embryonic development.

**Abstract:**

The purpose of this study was to investigate the effects and mechanisms of MitoQ on the IVM of culled bovine oocytes and subsequent embryonic development. The results revealed that in comparison to the control group (0 µmol/L), the IVM rate (*p* < 0.05) and subsequent blastocyst rate (*p* < 0.05) of the low-concentration 1 and 5 µmol/L MitoQ treatment group were increased. The level of ROS (*p* < 0.05) in the MitoQ treatment group was decreased in comparison to the control group. Additionally, the level of GSH, MMP, ATP, and mt-DNA in the MitoQ treatment group was increased (*p* < 0.05) in comparison to the control group. The expression level of BAX was decreased (*p* < 0.05) in the MitoQ treatment group, and the BCL2, DNM1, Mfn2, SOD, and CAT were increased (*p* < 0.05). In conclusion, MitoQ improved mitochondrial dysfunction, increased mitochondrial activity during IVM, and reduced oxidative stress, resulting in increased IVM rates and subsequent embryonic development from culled cows.

## 1. Introduction

In vitro maturation (IVM) is crucial for assisted reproductive technologies, including ovum pick-up (OPU), in vitro produced (IVP), and cloned embryos, which is an important step in IVP. Utilizing ovarian resources from selected culled cows or OPU from fat heifers, the IVM of the cumulus–oocyte complexes (COCs) in combination with in vitro fertilization (IVF) and embryo transfer (IVM-IVF-ET) is replacing the traditional technique of multiple ovulation and embryo transfer (MOET), which preserves the genes of the high genetic value of the cow [1,2]. Currently, OPU is less prevalent due to technical difficulties, numerous cows are eliminated from the herd each year culled cows and enhancing their IVM are crucial for advancing the IVP system. However, cull cows are mainly culled for reasons such as old age and impaired fertility, both of which are important factors affecting the quality of their oocytes. Previous studies indicate that mitochondrial DNA (mt-DNA) and adenosine triphosphate (ATP) levels are lower in oocytes and embryos of senescent cows compared to young cows, which affects the meiotic process and leads to chromosomal abnormalities [3]. COCs lose the antioxidant protection of the in vivo follicular environment and are exposed to in vitro conditions that inhibit the development of COCs. The maturation rate of COCs from slaughterhouse ovaries is significantly lower than that of in vivo oocytes [4,5]. In addition, bovine oocytes are cultured under high oxygen conditions, which can cause pre-maturation aging and affect oocyte nuclear and cytoplasmic maturations due to excessive accumulation of reactive oxygen species (ROS) [6,7].

Oxidative stress (OS) represents a significant factor affecting oocyte developmental potential, arising from an imbalance between ROS production and the antioxidant mechanism, leading to insufficient total antioxidant capacity to scavenge excess ROS [8]. In vitro conditions elevate ROS levels by exposing oocytes to an oxidative environment and separating them from the protective antioxidant environment of the follicle [9]. Mitochondria are essential for oocyte development and maturation, but they are a major source of ROS and vulnerable to OS. ROS accumulation can lead to intracellular DNA damage and oxidative modification of proteins, resulting in mitochondrial damage and cell death [10]. Therefore, adding antioxidants to bovine oocytes during IVM can mitigate OS damage. Several antioxidants, such as resveratrol [11,12], melatonin [13,14], and L-carnitine [15] have been shown to improve oocyte quality. Since most exogenous antioxidants are difficult to work inside the cell. Thus, mitochondria-targeted antioxidants show greater potential than non-targeted antioxidants in scavenging ROS produced by mitochondria.

The mitochondria-targeted antioxidant mitoquinol (MitoQ) is formed from a triphenylphosphine cation (TPP+) covalently bonded to the respiratory chain complex, species II coenzyme Q10, in the structure of a ten-carbon-atom aliphatic chain [16]. Its mechanism of action is mainly due to the difference in membrane potential and negative potential between the exterior mitochondrial membrane and the inner gap of mitochondria, the interior mitochondrial membrane and the interior matrix, the TPP+ part of its own higher lipid solubility and positive electricity. Therefore, it can serve as a carrier for the transmembrane transport of certain nuclear macromolecules that counteract OS from the mitochondrion to the mitochondrial matrix, helping to remove excessive ROS [17,18]. The respiratory chain is the carrier of electron transfer, and the hydrogen transmitter in the electron transfer of coenzyme Q10 plays an indispensable role in the respiratory chain but is also an essential component of mitochondrial synthesis of ATP. Studies have shown that reduced Coenzyme Q10 can remove a variety of free radicals from the intracellular, stabilize calcium ion balance channels, inhibit or reduce the OS of macromolecules such as proteins and DNA, and play an antioxidant role [19]. MitoQ accumulates into the mitochondria and is degraded into a form of ubiquinone with antioxidant activity. A portion of ubiquinone can be selectively inserted into the hydrophobic interior of the membrane; the mitochondrial matrix is the main absorption site so that MitoQ can continue to clear the active free radical series, thereby protecting mitochondria and improving the ability to resist OS damage. Subsequently, under the catalytic action of mitochondrial respiratory chain complex II, MitoQ is continuously reduced to ubiquinol with antioxidant activity [20].

MitoQ is an antioxidant with specific effects on mitochondria. Modifying the redox signaling pathway can reduce OS and protect mitochondrial function and has been successfully applied in many preclinical models of OS and apoptotic side effects [21,22]. Previous studies have shown that MitoQ prevents oxidative damage to many different tissues in rodents [23], alleviates neuropathic pain [24], and has a protective effect against diabetic nephropathy and pressure overload-induced cardiac damage [25,26]. MitoQ has a certain preventive and therapeutic effect on diseases related to the imbalance of the antioxidant system, OS, and mitochondrial dysfunction.

The above findings suggest that MitoQ may alleviate the age-induced decline in oocyte mitochondrial function by maintaining normal activity and reducing OS. Therefore, this study explored the effects of adding MitoQ to the maturation culture medium on IVM and subsequent embryonic development in oocytes. First, we examined the IVM rate and the subsequent blastocyst rate. Subsequently, we measured changes in ROS level, glutathione (GSH) level, mitochondrial membrane potential (MMP) level, ATP level, and mt-DNA copies number level in mature oocytes and analyzed expression alterations of antioxidant and mitochondria-related genes.

## 2. Materials and Methods

The MitoQ used in this study was purchased from Cayman Chemical (No. 89950, Ann Arbor, MI, USA). The compound MitoQ was dissolved in DMSO to 1 mmol/L stock solution. It was then diluted in IVM medium to final concentrations of 1, 5, and 10 µmol/L, respectively. IVM and IVC mediums were purchased from HARIOMED (Guangzhou, China). Chemicals were purchased from Sigma Aldrich (St. Louis, MO, USA) unless otherwise stated.

### 2.1. COCs Collection and In Vitro Maturation (IVM)

Bovine ovaries collected from cull cows at local slaughterhouse and delivered to laboratory in less than 4 h. The ovaries were cleaned more than 3 times in normal saline at 38.5 °C to wash away the blood from the ovaries. Follicular fluid was withdrawn from 2 to 8 mm follicles using an 18-G needle on a 10 mL single-use syringe and transferred to a centrifuge tube for 10 min. Then, the sediment at the bottom of the centrifuge tube was transferred to a Petri dish containing DPBS. Observing under a stereomicroscope, we selected COCs with minimum of three layers of dense cumulus cells for IVM.

The selected COCs were cleaned minimum of three times in an IVM medium. Thereafter, the group of 10 COCs was randomly seeded into 50 µL micro drops of IVM medium. Four different concentrations of MitoQ (0, 1, 5, 10 µmol/L) were used, 0 µmol/L as control group. The COCs were cultured in a 5% CO_2_ incubator at 38.5 °C for 22–24 h.

### 2.2. Parthenogenetic Activation (PA) and Embryo In Vitro Culture (IVC)

After IVM for 22–24 h, COCs were treated with 0.01% hyaluronidase to remove cumulus cells. Mature oocytes with first polar body were selected under stereoscope and washed in DPBS 2–3 times. Parthenogenetic activation was performed chemically using the (7% ethanol + 6-DMAP) method. Mature oocytes were transferred to IVC medium with 7% ethanol for 5 min. Then, the oocytes were transferred to IVC medium with 2 mmol/L 6-DMAP for 3 h in incubator. Finally, the oocytes were transferred to IVC medium in a 5% CO_2_ incubator at 38.5 °C. Cleaved embryos were observed at Day 2 (after 48 h of activation), and the blastocyst embryos were observed at Day 7 or Day 8 after activation.

### 2.3. Assessment of ROS Level

Following the manufacturer’s instructions, ROS level was measured by using a ROS detection kit (Beyotime, Shanghai, China). After maturing for 22–24 h, COCs were treated with 0.01% hyaluronidase to remove cumulus cells. The matured oocytes were selected and washed three times in DPBS. Then, the oocytes were incubated in DPBS containing 10 µmol/L DCFHDA dye for 20 min at 38 °C in the dark. After incubation, the oocytes were washed 3 times with DPBS. DCFHDA dye was excited at 488 nm and photographed under a fluorescence microscope (ZEISS, Oberkochen, Germany). Image J (1.54 i) software was used to analyze the green fluorescence intensity of ROS level.

### 2.4. Assessment of GSH Level

Following the manufacturer’s instructions, GSH levels were measured by using the Cell-tracker Blue CMF2HC Dye (MedChemExpress, Monmouth Junction, NJ, USA). After maturing for 22–24 h, COCs were treated with 0.01% hyaluronidase to remove cumulus cells. The matured oocytes were selected and washed three times in DPBS. Then, the oocytes were incubated in DPBS containing 10 µmol/L CMF2HC dye for 20 min at 38 °C in the dark. After incubation, the oocytes were washed 3 times with DPBS. CMF2HC dye was excited at 400–405 nm and photographed under a fluorescence microscope (ZEISS, Oberkochen, Germany). Image J software was used to analyze the blue fluorescence intensity of GSH level.

### 2.5. Assessment of MMP Level

Following the manufacturer’s instructions, the MMP levels were measured by using the TMRE detection Kit of MMP (Beyotime, Shanghai, China). After maturing for 22–24 h, COCs were treated with 0.01% hyaluronidase to remove cumulus cells. The matured oocytes were selected and washed three times in DPBS. Then, the oocytes were incubated in detection solution containing TMRE for 20 min at 38 °C in the dark. After incubation, the oocytes were washed 3 times with DPBS. TMRE dye was excited at 550 nm and photographed under a fluorescence microscope (ZEISS, Oberkochen, Germany). Image J software was used to analyze the red fluorescence intensity of MMP level.

### 2.6. Assessment of ATP Level

Following the manufacturer’s instructions, the ATP level was measured by using the ATP detection kit (Beyotime, Shanghai, China). After maturing for 22–24 h, COCs were treated with 0.01% hyaluronidase to remove cumulus cells. The matured oocytes were selected and washed three times in DPBS. Then, 10 matured oocytes were placed in each group, and the oocytes were fully lysed with 200 µL of cell lysis buffer. After lysis, the solution was centrifuged at 4 °C for 5 min at 12,000 rpm. The supernatant was collected and stored on ice until detection. Finally, 100 µL of ATP assay solution was added to each well of a 96-well plate and waited for 5 min. Then, 20 µL of sample supernatant was added to each well and mixed. Detecting luminescence used a luminometer (Spark; Tecan, Groedig, Austria, G.m.b.H) and analyzed ATP level according to the standard curve.

### 2.7. DNA Extraction and Analysis of Mitochondrial DNA (mt-DNA) Copies Number by qPCR

Following the manufacturer’s instructions, DNA was extracted from 10 matured oocytes of each group from control and treatment groups separately by using a FastPure Cell/Tissue DNA Isolation Mini Kit (Vazyme, Nanjing, China). qPCR was performed by adding 1 µL the previously extracted DNA to a 5 µL mixture of Taq Pro Universal SYBR qPCR Master Mix (Vazyme, Nanjing, China), forward and reverse primers (10 µM), the RNase-free water, the final volume of 10 µL. The qPCR cycle was set to 95 °C for 30 s, then 95 °C for 5 s, and 60 °C for 30 s each for 40 cycles. The primers used for qPCR were designed using the Primer BLAST software of the National Center for Biotechnology Information (NCBI) (https://www.ncbi.nlm.nih.gov/tools/primer-blast/, accessed on 13 September 2024), as shown in Table 1. In addition, the use of mt-ND1 as a target gene in this study was in reference to a previous study [27]. Using β-actin as reference gene, the relative expression level of mt-DNA copies number was measured by qPCR and calculated based on the 2^−∆∆Ct^ method.

### 2.8. Analysis of Gene Expression by qPCR

Following the manufacturer’s instructions, cDNA was directly synthesized from 5 matured oocytes of each group from control and treatment groups separately by using a QuantAccuracy RT-RamDA cDNA Synthesis Kit (TOYOBO, OSAKA, OSAKA, Japan). The qPCR reagent was 1 µL of cDNA added to 5 µL of Taq Pro Universal SYBR qPCR Master Mix (Vazyme, Nanjing, China), forward and reverse primers (10 µM), RNase-free water, the final volume of 10 µL. The qPCR cycle was set to 95 °C for 30 s, then 95 °C for 5 s, and 60 °C for 30 s each for 40 cycles. The primers are shown in Table 1. Using GAPDH as reference genes, the relative expression levels of target genes were measured by qPCR and calculated based on the 2^−∆∆Ct^ method.

### 2.9. Statistical Analysis

All experiments were statistically analyzed using at least 3 groups of biological replicates. Data were analyzed and graphed using GraphPad Prism 9.0 software. Comparisons between groups were made using one-way analysis of Tukey’s or Dunnett’s post-test, the results were expressed as mean ± standard error (mean ± SEM). In this experiment, values of *p* < 0.05 were considered to indicate statistical significance.

## 3. Results

### 3.1. Effect of MitoQ on IVM and Embryonic Developmental Capacity

The COCs after 22–24 h of IVM are shown in Table 2. The first polar body extrusion (PBE) of oocytes was used as the standard for maturation. The proportion of PBE rates of MitoQ-treated groups (1 and 5 µmol/L) was increased (*p* < 0.05) compared to the control group (0 µmol/L). There was no significant difference (*p* > 0.05) between the proportion of the PBE rate in the 10 µmol/L group and the 0 µmol/L group.

**Figure 1:**
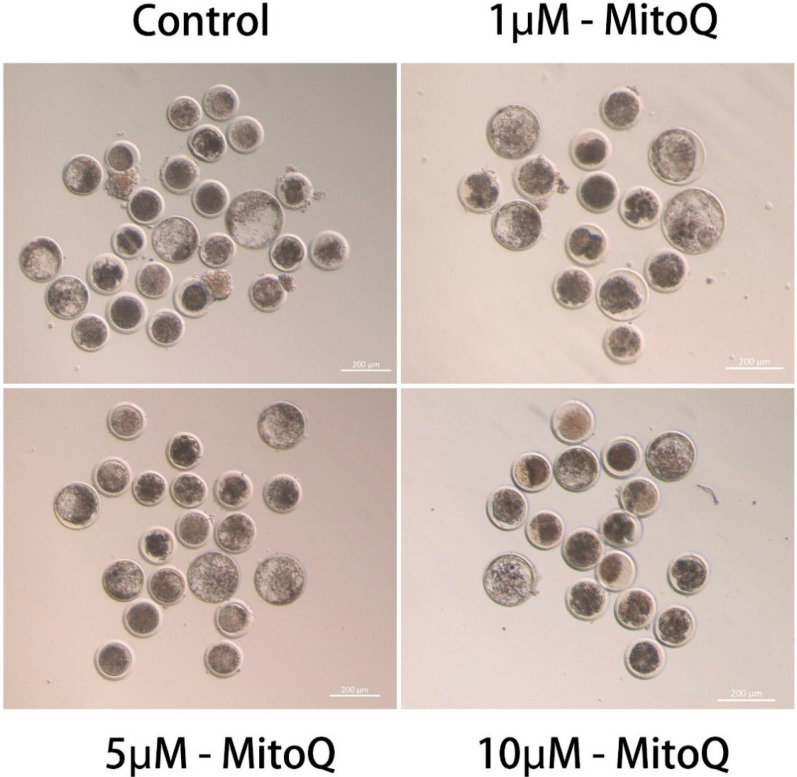
Influences of MitoQ on subsequent embryonic development and quality. Representative images of blastocyst development on day 7 for control group and (1, 5, 10 µmol/L) MitoQ treatment groups. Scale bar: 200 µm.

In addition, the further assessment of the subsequent developmental potential of PA embryos and the results are shown in Table 3. The proportion of cleavage rate of MitoQ-treated groups (1, and 5 µmol/L) was increased (*p* < 0.05) compared to the control group (0 µmol/L). The 1 and 5 µmol/L MitoQ treated groups resulted in increased blastocyst rate (*p* < 0.05). However, there was no significant difference (*p* > 0.05) between the proportion of cleavage and blastocyst rate in the 10 µmol/L group and the 0 µmol/L group (Figure 1). According to these findings, MitoQ can improve the IVM of culled bovine oocytes and subsequent embryo development. Therefore, we used the 5 µmol/L MitoQ group as the experimental group for the following experiments.

### 3.2. Influences of MitoQ on ROS and GSH Level in Bovine Oocytes

**Figure 2:**
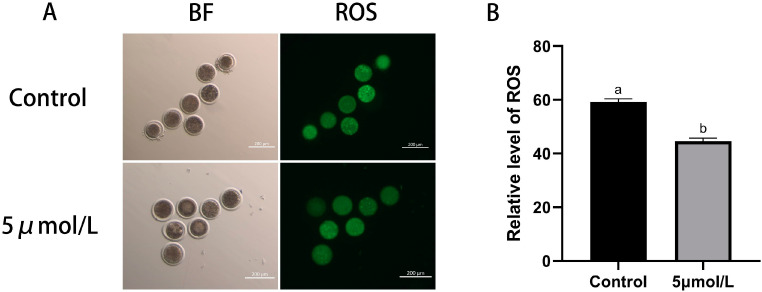
Influences of MitoQ on the ROS level in matured bovine oocytes. (**A**) Representative images of ROS (green) staining of oocytes in control group and MitoQ treatment group. Scale bar: 200 µm. (**B**) Analysis of ROS fluorescent intensity data. All experiments were performed in at least three replications, and the data statistics were represented by mean ± SEM. Values (a, b) with different superscripts in the columns are significantly different (*p* < 0.05).

In this study, we explored how MitoQ improves the IVM of culled bovine oocytes by detecting the level of ROS and GSH in the oocyte oxidation–reduction system of oocytes, thereby increasing the maturation rate of oocytes. The results shown in Figure 2 indicate that the level of ROS in matured oocytes in the MitoQ-treated group was lower than those in the control group (*p* < 0.05). Thus, MitoQ was able to decrease ROS production in oocytes.

**Figure 3:**
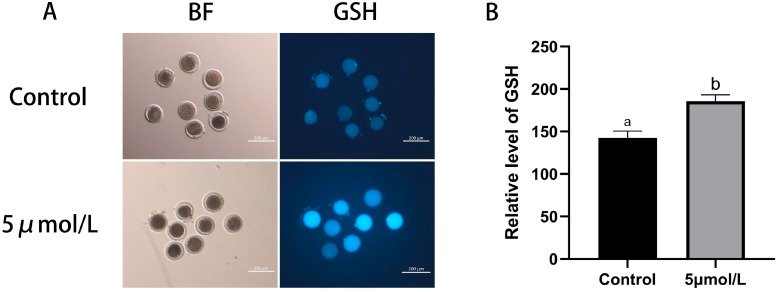
Influences of MitoQ on the GSH level in matured bovine oocytes. (**A**) Representative images of GSH (blue) staining of oocytes in control group and MitoQ treatment group. Scale bar: 200 µm. (**B**) Analysis of GSH fluorescent intensity data. All experiments were performed in at least three replications, and the data statistics were represented by mean ± SEM. Values (a, b) with different superscripts in the columns are significantly different (*p* < 0.05).

This experiment also detected the level of GSH production in oocytes; the results are shown in Figure 3. The level of GSH in matured oocytes MitoQ treated group was higher than those in the control group (*p* < 0.05). The findings suggest that MitoQ can enhance the antioxidant capacity of culled bovine oocytes, resulting in the reduction of the adverse effects of ROS on oocytes.

### 3.3. Influences of MitoQ on MMP Level in Bovine Oocytes

**Figure 4:**
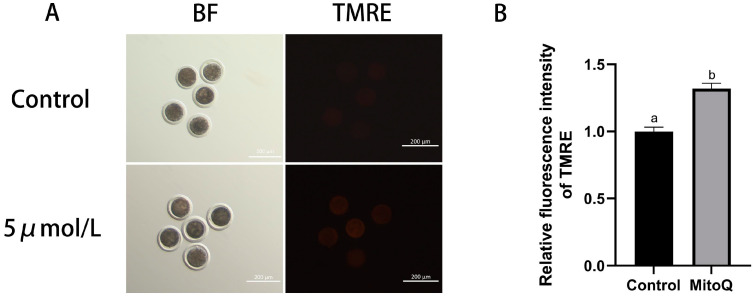
Influences of MitoQ on the MMP level in matured bovine oocytes. (**A**) Representative images of MMP (red) staining of oocytes in control group and MitoQ treatment group. Scale bar: 200 µm. (**B**) Analysis of MMP fluorescent intensity data. All experiments were performed in at least three replications, and the data statistics were represented by mean ± SEM. Values (a, b) with different superscripts in the columns are significantly different (*p* < 0.05).

Assessment of mitochondrial function by detection of MMP in culled bovine oocytes. The results shown in Figure 4 indicate that the level of MMP in matured oocytes in the MitoQ-treated group was higher than those in the control group (*p* < 0.05). The results showed that MitoQ improved the quality and mitochondrial function of oocytes and attenuated the mitochondrial damage caused by the OS environment, thus promoting the maturation of oocytes.

### 3.4. Influences of MitoQ on ATP and mt-DNA Copies Number Levels in Bovine Oocytes

**Figure 5:**
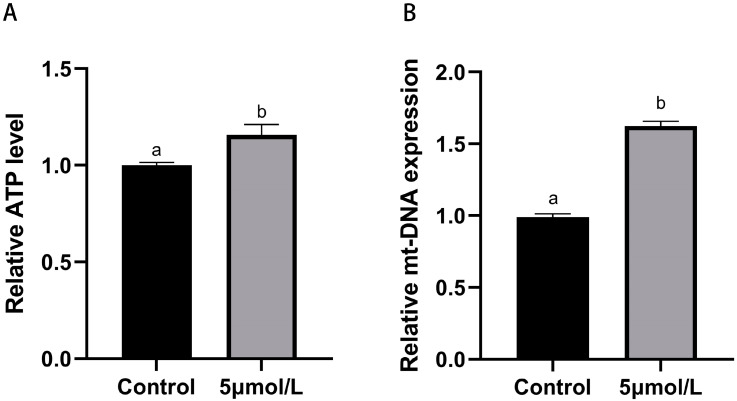
Influences of MitoQ on mitochondrial function in matured bovine oocytes. (**A**) Quantitative analysis of ATP content level. (**B**) Quantitative analysis of mt-DNA copies number level. All experiments were performed in at least three replications, and the data statistics were represented by mean ± SEM. Values (a, b) with different superscripts in the columns are significantly different (*p* < 0.05).

Mitochondria are the most important organelles in the maturation process of oocytes; their activity directly influences the developmental potential of the oocytes. To study whether MitoQ improves mitochondrial function in culled bovine oocytes, we detected the level of ATP and mt-DNA copies number in oocytes. As shown in Figure 5, the levels of ATP and mt-DNA copies number in matured oocytes MitoQ treated group were higher than those in the control group (*p* < 0.05).

### 3.5. Influences of MitoQ on Related Genes Expression

**Figure 6:**
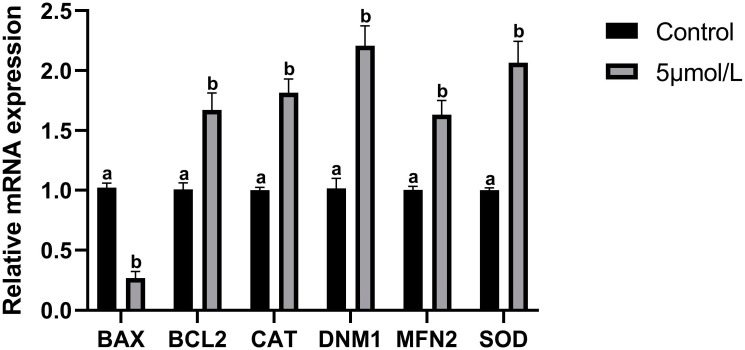
Influences of MitoQ on antioxidant-related and mitochondria-related genes in matured bovine oocytes. qPCR quantitative analysis of mRNA expression of apoptosis-related genes (BAX, BCL2), antioxidant-related genes (CAT, SOD), and mitochondria-related genes (DNM1, MFN2). All experiments were performed in three replications, and the data statistics were represented by mean ± SEM. Values (a, b) with different superscripts in the columns are significantly different (*p* < 0.05).

The results of mRNA expression levels of apoptosis-related genes in culled bovine oocytes are shown in Figure 6. The relative mRNA expression level of the pro-apoptotic gene BAX was downregulated in the MitoQ-treated group than those in the control group (*p* < 0.05). Whereas those of the anti-apoptotic gene BCL2, antioxidant-related genes CAT and SOD1, and mitochondrial fusion protein-related genes DNM1 and MFN2 were upregulated (*p* < 0.05).

## 4. Discussion

In this study, we investigated the effects of MitoQ on the IVM of culled bovine oocytes and subsequent embryonic development. The results showed that adding a low concentration of MitoQ (1 and 5 µmol/L) to the IVM medium significantly increased maturation rates. In addition, further PA experiments confirmed that MitoQ enhances the development of subsequent embryos into blastocysts, indicating that MitoQ has a good performance of OS regulating effect. Maintaining a stable balance between pro-oxidants and antioxidants is essential for obtaining quality oocytes and embryos, but high concentrations of antioxidants can disrupt the meiotic process [28]. High concentrations of MitoQ decrease maturation rates in mouse oocytes [29]. The cationic carrier (TPP+) of MitoQ causes cation overload in mitochondria, affecting mitochondrial function, resulting in reduced cleavage and blastocyst rates with 1 μmol/L MitoQ during IVM [30]. In contrast, we found that adding 1 and 5 µmol/L MitoQ increased maturation rates, while 10 µmol/L MitoQ had no effect on IVM. This may be due to mitochondrial dysfunction in cull cow oocytes, leading to an imbalance in intracellular Ca^2+^ homeostasis and metabolic impairment. Reduced mitochondrial Ca^2+^ transfer impairs mitochondrial metabolism, resulting in defective ATP production [31]. TPP+ increases Ca^2+^ levels in mitochondria by inhibiting Ca^2+^ efflux from organelles [32]. In our study, TPP+ may increase Ca^2+^ levels in mitochondria without impairing mitochondrial function due to cation overload. Compared to oocytes with normal mitochondrial function, MitoQ may be more effective for those with dysfunction. Because this study was limited by ovarian resources, it was not possible to do more lower concentration gradient groups of experiments. Therefore, it can only be proved that 1 and 5 µmol/L MitoQ promotes IVM, but the optimal concentration of MitoQ addition needs to be explored further.

In healthy oocytes, the relationship between the regulation of mitochondrial activity and ROS production is regulated [33,34]. Free radicals are normal metabolic products of the cells, and under normal conditions, their production and removal maintain a dynamic balance within a certain range [35]. OS results from destabilized intracellular stability, leading to excessive free radical accumulation. Our results showed that the addition of the MitoQ treatment group significantly reduced ROS levels in oocytes. Consistent with previous studies, MitoQ attenuates liver fibrosis in mouse hepatocytes by inhibiting OS [36]. MitoQ ameliorates endogenous oxidative DNA damage in peripheral mononuclear cells exposed to exogenous ROS [37]. MitoQ effectively enters the mitochondrial matrix and removes excess free radicals, thereby reducing their concentrations [38].

As a high-efficiency antioxidant, GSH binds peroxides and free radicals during redox processes, reducing their damage to oocytes [39]. Therefore, GSH is used as a pivotal parameter to assess cytoplasmic maturation [40]. A correlation between the level of ROS accumulation and GSH level has been shown in previous studies [41]. The decrease in the production of GSH is accompanied by an increase in ROS production, which can inhibit oocyte development at the GV stage [42]. Thus, increasing intracytoplasmic GSH levels in early development and mature oocytes protects oocytes and subsequent embryos from ROS damage [43]. Our results show that adding MitoQ, which increases GSH level and decreases ROS level, reduces the effects of OS on oocytes and enhances their ability for IVM.

As an important energy supplier of cells, mitochondria are indispensable in the process of IVM [44]; it likewise plays a pivotal factor in the subsequent development of the embryo [45]. Its function can be reflected through the MMP level [46]. MMP plays a vital function in the production of ATP production and the maintenance of metabolic regulation during oocyte maturation [47,48]. Most of the ATP in oocytes is generated from mitochondrial electron transport chain-coupled oxidative phosphorylation (OXPHOS) [49]. However, ROS are potentially harmful byproducts of the OXPHOS process. Increased ROS levels disrupt the meiotic spindle, resulting in reduced ATP level and mt-DNA copies number, leading to mitochondrial dysfunction [50,51]. In addition, the quality and quantity of mitochondria are recognized as the major indicators of oocyte quality because the low ATP level and mt-DNA copy number result in low developmental competence of oocytes [52,53]. MitoQ protects MMP from OS damage in a lipopolysaccharide–peptidoglycan model of sepsis [54]. MitoQ increases sperm survival after thawing in yellow catfish by increasing mitochondrial membrane integrity [55]. In addition, MitoQ ameliorates spindle abnormalities and chromosomal misalignment in aged mice oocytes during IVM and improves IVM rate and spindle chromosome alignment in human oocytes [56]. The ovaries sampled in this study were from culled cows in the abattoir. Our results showed that the MMP level, ATP level and mt-DNA copies number in matured oocytes MitoQ treated group were significantly higher than those in the control group. Therefore, the MitoQ treatment group may benefit from increased MMP, ensuring mitochondrial stability and energy production for mitosis, which improves spindle abnormalities and chromosome disorders in culled bovine oocytes. It also improved the antioxidant capacity of oocytes, promoting their quality. The ability of MitoQ to reverse senescence in oocytes from older cows positively impacts the IVP system by utilizing ovarian resources from high-yielding culled cows or OPU techniques.

OS can induce autophagy and early apoptosis [57]. In the mitochondria-mediated apoptosis pathway, BAX is activated by abnormal oocyte signaling, disrupts BCL-XL expression, and induces apoptosis [58,59]. We detected apoptosis-related genes BAX and BCL2 in oocytes after IVM; the results showed that MitoQ significantly increased the expression of BCL2 and decreased the expression of BAX, indicating that MitoQ could reduce the apoptosis of oocytes. In addition, SOD is part of the enzymatic defense system against OS by converting superoxide radical anions to H_2_O_2_. CAT is expressed in most cells, organs, and tissues. It is the enzyme involved in the reduction of H_2_O_2_ to water [60]. Our results showed that MitoQ significantly increased the expression of SOD and CAT, suggesting that the antioxidant effect of MitoQ in oocytes may be mainly attributed to its regulation of enzyme defense systems SOD1 and CAT. These results are consistent with previous studies [61,62].

The fission and fusion of mitochondrial membrane is an important guarantee for mitochondria to maintain normal function, we further detected the mitochondrial fission gene Dynamin-1 (DNM1) and fusion-related genes Mitofusin-2 (MFN2). DNM1 catalyzes the membrane fission of mitochondria in cells [63]. Deletion of the DNM1 gene leads to reduced mitochondrial division and inhibition of cell growth [64]. On the other hand, mitochondria are involved in the regulation of intracellular material metabolism, calcium homeostasis, cell proliferation, differentiation, and apoptosis through the mitochondrial fusion of MFN2. By coupling the endoplasmic reticulum and mitochondria, MFN2 participates in Ca^2+^ stabilization in oocytes, increases mitochondrial activity, and regulates oocyte development and maturation [65]. Our results showed that the relative expression levels of DNM1 and Mfn2 increased significantly in the MitoQ treatment group. Previous studies have shown that MitoQ improves mitochondrial biosynthesis by increasing the MFN2 expression [66]. Under OS, MitoQ reverses mitochondrial damage and maintains MFN2 at a normal expression level [24]. These results indicate that MitoQ can enhance the ability of mitochondrial fission and fusion.

## 5. Conclusions

In conclusion, we found that MitoQ can improve the intracellular antioxidant system by restoring mitochondrial metabolic function through its targeted antioxidant properties, eliminating excessive free radicals, and stabilizing the intracellular environment. It can reduce OS generation, alleviate oxidative damage, and improve the development of culled bovine oocytes. MitoQ provides rational use of oocyte resources from culled cows and provides a new attempt to optimize the IVM-IVP system in the future.

## Figures and Tables

**Table 1:** List of primer pairs used for qPCR.

Gene	Primer Sequence (5′-3′)	GenBank Accession No.	Product Size (bp)
BAX	F: TGAGCAGATCATGAAGACAGGG	NM_173894.1	241
	R: CGCCACTCGGAAAAAGACCT		
BCL2	F: AGGCAGGCGATGAGTTTGAA	NM_001077486.2	159
	R: AGAAAGAGGGCCAAATGCGA		
CAT	F: GAGGAAACGCCTGTGTGAGA	NM_001035386.2	116
	R: GGATGCGGGAGCCATATTCA		
DNM1	F: CTTCACACCTGACCTCGCTT	NM_001076820.1	117
	R: TTTCTGATGGTGGCCGTGAG		
MFN2	F: GGGCTCAGAGGAGAAGAGGA	NM_001190269.1	72
	R: AGCTGTTCGTCCTGGTGAAG		
SOD	F: AAAACACGGTGGGCCAAAAG	NM_174615.2	186
	R: TTTCCACCTCTGCCCAAGTC		
GAPDH	F: CGCCATCAATGACCCCTTCA	NM_001034034.2	71
	R: TGCCGTGGGTGGAATCATAC		
mt-ND1	F: CAGTGAGAATGCCCTCTAGG		225
	R: TTTACGCCGTACTCCTGT		
β-actin	F: AAGATCAAGATCATCGCGCC	NM_173979.3	174
	R: GTAACGCAGCTAACAGTCCG		

F = forward; R = reverse; bp = base pairs.

**Table 2:** Effects of MitoQ on IVM of bovine oocyte.

Conc. of MitoQ (μM)	NO. of COCs	NO. of Oocytes Matured (PBE)	PBE Rate (% ± SEM)
0	294	201	68.62 ± 1.17 ^a^
1	268	194	72.16 ± 0.64 ^b^
5	311	226	72.54 ± 0.90 ^b^
10	247	169	68.40 ± 0.57 ^a^

Data are shown in mean ± SEM based on the percentages of each replicate. Values (a, b) with different superscripts in the columns are significantly different (*p* < 0.05).

**Table 3:** Effects of MitoQ on subsequent parthenogenetic embryo culture.

Conc. of MitoQ (μM)	NO. of PA Embryos	Cleavage (% ± SEM)	Blastocyst Day7 (% ± SEM)
0	104	77 (74.12 ± 0.83) ^a^	27 (25.88 ± 0.83) ^a^
1	113	87 (76.95 ± 1.09) ^b^	35 (30.99 ± 0.44) ^b^
5	107	83 (77.78 ± 0.93) ^b^	33 (30.79 ± 0.51) ^b^
10	99	72 (72.50 ± 0.77) ^a^	25 (25.03 ± 0.75) ^a^

Data are shown in mean ± SEM based on the percentages of each replicate. Values (a, b) with different superscripts in the columns are significantly different (*p* < 0.05).

## Data Availability

Data are contained within the article.
